# Editorial: Pain and pain-related neuropsychiatric disorders: from mechanistic insights to innovative therapeutic strategies

**DOI:** 10.3389/fphar.2025.1593890

**Published:** 2025-03-25

**Authors:** Rosmara Infantino, Fabio Turco, Francesca Guida, Álvaro Llorente-Berzal

**Affiliations:** ^1^ Pharmacology and Therapeutics, School of Medicine, University of Galway, Galway, Ireland; ^2^ Centre for Pain Research, University of Galway, Galway, Ireland; ^3^ Galway Neuroscience Centre, University of Galway, Galway, Ireland; ^4^ Cannabiscientia SA, Zurich, Switzerland; ^5^ Section of Pharmacology L. Donatelli, Department of Experimental Medicine, University of Campania Luigi Vanvitelli, Naples, Campania, Italy; ^6^ Department of Physiology, Faculty of Medicine, Autonomous University of Madrid, Madrid, Madrid, Spain

**Keywords:** chronic pain, pain mechanisms, pain models, pain therapies, neuropsychiatric disorders

## Introduction

Chronic pain is a pervasive health challenge, affecting approximately 30 per cent of the world’s population and imposing a significant personal and socio-economic burden. In addition to its sensory components, chronic pain has important affective and cognitive dimensions and is closely linked to neuropsychiatric complications, including anxiety, depression and cognitive dysfunction. Despite significant advances in basic and clinical research, the management of chronic pain remains inadequate, as translation from mechanistic findings to clinical applications faces persistent barriers. Similarly, clinical observations often lack mechanistic context by not being adequately integrated with basic research. The aim of this special Research Topic was to fill this gap by promoting a synergistic integration of preclinical and clinical trends towards new pharmacological approaches or strategies to improve pain management. Particular attention has been given to sex and gender differences in pain perception. In addition, with a view to sustainability, special emphasis was placed on mechanistic and clinical studies on substances of natural origin.

## Mechanistic insights charting a path for translational impact

A fundamental step toward improving chronic pain treatment lies in deciphering its underlying neurobiological mechanisms with the use of well-characterised animal models. In this regard, the preclinical study by Di Marino et al. extends behavioural characterisation of the paclitaxel model of chemotherapy-induced neuropathic pain in rats of both sexes, and sheds light on the status of the endocannabinoid system in this model. Their work lays a solid foundation for potential targeting of endocannabinoid signalling as a novel therapeutic strategy. The endocannabinoid system is explored also in the work by Yuan et al. They explore novel pathways, including the role of VD-hemopressin (α) and RVD-hemopressin (α) in electroacupuncture-mediated pain relief, highlighting a role for the cannabinoid type 1 (CB_1_) receptor. Qu et al. further elaborate on neuropathic pain mechanisms, demonstrating the efficacy of the plant Dahuang Fuzi, whose decoction has been used for millennia in traditional Chinese medicine, in inhibiting TNF-α and PI3K-AKT signalling in the chronic constriction injury model (CCI) of neuropathic pain. Novel insight on thermal nociception is offered by Engel et al. Their work investigates the interplay between sodium-activated potassium channel Slick (Kcnt2) and the transient receptor potential melastatin (TRPM3), which is highly co-expressed in specialised heat-sensitive neurons, highlighting possible new pharmacological targets.

## Clinical studies and systematic reviews: toward personalised medicine

Clinical studies included in this Research Topic represent a significant contribution to developing patient-centered interventions.

Baeumler et al. contribute to the ongoing debate on predicting acupuncture analgesic response in relation to high temporal summation (TS), reporting that acupuncture effectively reduces pain in patients with chronic non-specific low back pain (LBP) regardless of TS levels, supporting its use as a viable treatment for a broader population. The clinical trial by Scuteri et al. presents compelling evidence on the efficacy of a nanoparticle formulation of bergamot essential oil (NanoBEO) in managing both pain and agitation in patients with severe dementia, highlighting a natural-derived alternative to conventional analgesics in these complex patients already undergoing polypharmacotherapy. Takahashi et al. further explore the neuropsychiatric component in ADHD-comorbid nociplastic pain, highlighting the functional hyperactivity of the praecuneus, a key brain region involved in self-referential thinking, attention, and pain processing. A focus on the affective component, including the pain catastrophizing, of menstrual pain is given by Hsu et al. They investigate how catechol-O-methyltransferase (COMT) Val158Met polymorphisms influence the top-down pain modulation within the reward system, significantly contributing to individual differences in pain regulation. Papa et al. provide crucial long-term data on buprenorphine transdermal patches, demonstrating its sustained analgesic effects with reduced side effects and reduced risk of addiction compared to other opioids, reinforcing its role as a viable option for chronic pain management. Finally, the metabolomic profile of the novel anti-NGF antibody DS002 is reported by Jin et al. This phase I study shows how cartilage and bone metabolism are not significantly affected by treatment with the antibody, suggesting that DS002 may offer a safer alternative to existing anti-NGF analgesics with fewer joint-related adverse effects.

Systematic reviews also play a pivotal role in consolidating knowledge and guiding future research.

The meta-analysis by Peng et al. on levetiracetam for paediatric migraine prophylaxis offers valuable insights into its efficacy and underscores the need for further exploration on its safety profile. Starting from a case report, the scoping review by Peraire et al. on ziconotide-related psychosis raises awareness of the neuropsychiatric implications of this treatment. A mechanism related to the blockade of N-type voltage-gated calcium channels in glutamatergic and GABAergic neurons is hypothesised, highlighting the critical need to balance pain relief and patient safety.

## Reviews and future directions

As the field evolves, reviews provide a comprehensive synthesis of emerging trends and future perspectives. The discussion by Yang et al. on mirogabalin as a novel calcium channel α2δ ligand underscores its promising role in the treatment of neuropathic pain and related comorbidities. Finally, Kasahara et al. explore the potential of ADHD medications for chronic pain management, opening new interdisciplinary avenues.

Looking ahead, advancing personalised medicine, exploring sex-specific differences, and targeting novel pharmacological pathways will refine pain treatment. Integrating mechanistic insights with clinical applications is essential for addressing pain and its neuropsychiatric complexities. This Research Topic highlights the crucial link between basic and clinical research, pushing us closer to effective solutions for chronic pain and associated disorders.

